# Normal SUV Values Measured from NaF18- PET/CT Bone Scan Studies

**DOI:** 10.1371/journal.pone.0108429

**Published:** 2014-09-25

**Authors:** Aung Zaw Win, Carina Mari Aparici

**Affiliations:** 1 Department of Radiology, San Francisco VA Medical Center, San Francisco, California, United States of America; 2 Department of Radiology, University of California San Francisco (UCSF), San Francisco, California, United States of America; Genentech, United States of America

## Abstract

**Objectives:**

Cancer and metabolic bone diseases can alter the SUV. SUV values have never been measured from healthy skeletons in NaF18-PET/CT bone scans. The primary aim of this study was to measure the SUV values from normal skeletons in NaF18-PET/CT bone scans.

**Methods:**

A retrospective study was carried out involving NaF18- PET/CT bone scans that were done at our institution between January 2010 to May 2012. Our excluding criteria was patients with abnormal real function and patients with past history of cancer and metabolic bone diseases including but not limited to osteoporosis, osteopenia and Paget’s disease. Eleven studies met all the criteria.

**Results:**

The average normal SUV_max_ values from 11 patients were: cervical vertebrae 6.84 (range 4.38–8.64), thoracic vertebrae 7.36 (range 6.99–7.66), lumbar vertebrae 7.27 (range 7.04–7.72), femoral head 2.22 (range 1.1–4.3), humeral head 1.82 (range 1.2–2.9), mid sternum 5.51 (range 2.6–8.1), parietal bone 1.71 (range 1.3–2.4).

**Conclusion:**

According to our study, various skeletal sites have different normal SUV values. SUV values can be different between the normal bones and bones with tumor or metabolic bone disease. SUV can be used to quantify NaF-18 PET/CT studies. If the SUV values of the normal skeleton are known, they can be used in the characterization of bone lesions and in the assessment of treatment response to bone diseases.

## Introduction

SUV value is defined as the tissue concentration of tracer as measured by a PET scanner divided by the activity injected divided usually by body weight [1]. The uptake value is represented by pixel or voxel intensity value in the ROI of the image, which is then converted into the activity concentration. SUVs represent tissue activity within an ROI corrected for injected activity and body weight [2]. There is diminishing bone perfusion with ageing [3]. So, the SUV values can be different between the aged and adults. Several studies have pointed out that there is no such magic cut-off SUV value to label a finding either benign or malignant [4], [5]. On the other hand, Waterval et al wrote that SUV measurements in NaF18 PET/CT studies have the potential to be a diagnostic tool [6].

The usual imaging time is 45–60 min for NaF-18 PET studies. However, the tracer uptake in bone does not plateau for several hours [1]. SUV values vary depending on the organ of the body. Age related changes of the bone are more pronounced in some bone locations compared to others. For example, parietal and occipital bones show more age related changes compared to the rest of the skull [7]. It must be noted that blood flow varies among different bones. The uptake of NaF in the bone depends on the blood flow to the area, regional osteoblastic activity and on renal clearance [8]. Cancellous bone is less dense but with a higher surface area than cortical bone. It typically occupies the interior region of bones, is highly vascular and frequently contains bone marrow [9]. Cancellous bone forms only 20% of the bone mass but accounts for 80% of the bone turnover associated with remodeling [10]. Thus, cancellous bone can have more SUV uptake than cortical bone.

The diffusion of NaF into the bone leads to a slow exchange of fluoride ions with hydroxide ions of the hydroxyapatite crystals, eventually forming fluoroapatite, a process that begins rapidly but takes many days to weeks to complete [8]. The rapid uptake of 18F-fluoride occurs preferentially at sites of high osteoblastic activity where bone remodeling is greatest. Yet, the tracer can accumulate in both osteoblastic and osteolytic lesions [11]. F-18 ion has a high affinity for bone that leads to a large tissue to background ratio and hence good-quality images [2]. NaF18 has two-fold greater tracer accumulation in skeletal system compared with Tc99m-MDP [12]. Unlike Tc99m tracer, there is no protein binding for NaF18 and NaF18 has faster blood and renal clearance [12]. NaF18-PET/CT bone scan has less radiation exposure than Tc99m-MDP SPECT/CT [13]. Even-Sapir et al reported that NaF18-PET/CT has a sensitivity and specificity of 100% respectively for detecting prostate cancer metastases [14]. It is certainly more sensitive and more specific than Tc99m-MDP bone scan. Metastatic diseases can detect much earlier with NaF18-PET/CT than with Tc99m-MDP bone scan [15].

NaF 18 PET/CT can detect skeletal metastases of tumors that typically have low FDG avidity, such as thyroid cancer or renal cell cancer [16]. Not all malignant lesions are reliably identified due to variable rates of glucose metabolism, contributing to the overall limitation of FDG PET/CT [17]. Bone marrow can exhibit nonspecific widespread intense FDG uptake following recent chemotherapy and this can limit evaluation for osseous metastases [18]. FDG PET/CT can sometimes fail to detect metastasis to the bone [18]. Uptake of the fluoride tracer by the bone marrow is negligible. FDG can also bind nonspecifically to the surrounding soft tissue after radiation therapy in post-radiation myositis. This can mask the metastatic lesion in the bone. 18F-fluoride is unaffected by recent chemoradiation therapy because its uptake is in the mineral component of the bone [18]. No limitations to diet of physical activity are required for this exam, whereas, for FDG PET/CT, the patient has to limit physical activity to avoid increased FDG uptake by the muscles.

To our knowlwdge, no report has been published on SUV (standard uptake value) of bone in patients without history of cancer or metabolic bone disease. Studies have been done on patients with osteoporosis and patients with bone metastasis. In addition, research on SUV in NaF18 exams is very limited. The primary aim of this study was to report the SUV values from normal skeletons in NaF18-PET/CT bone scans.

## Methods

**Figure 1:**
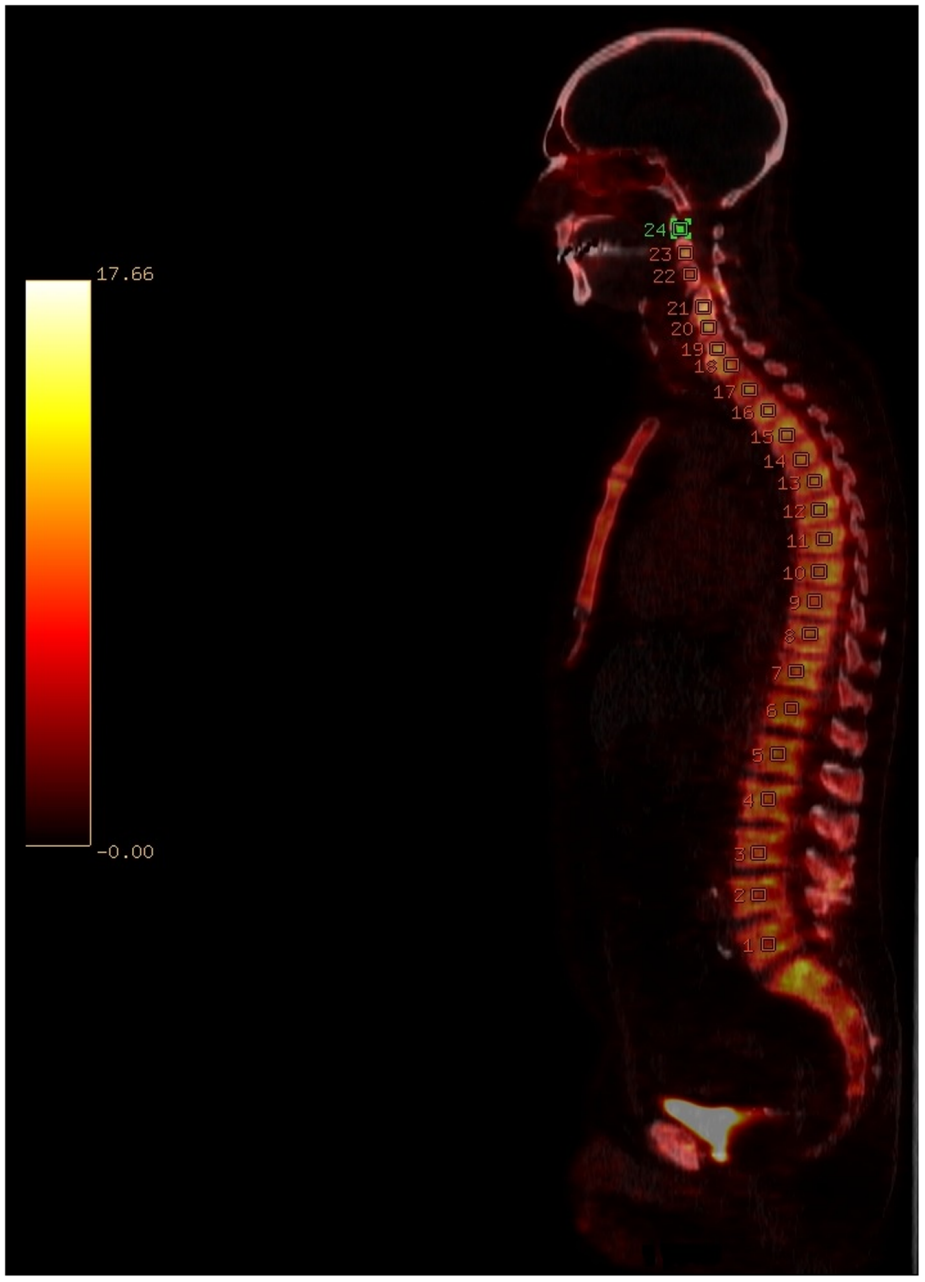
A fixed-size ROI (826 mm^3^) was placed on the vertebral bodies (C1-L5) to measure the SUV_max_ values of the spine.

This study was approved by the San Francisco VA Medical Center IRB. Patient information was anonymized and de-identified prior to analysis. We retrospectively reviewed all the NaF18 PET/CT bone scans done at our institution between January 1, 2010 and May 31, 2012. Our excluding criteria was patients with abnormal real function and patients with past history of cancer and metabolic bone diseases including but not limited to osteoporosis, osteopenia and Paget’s disease. We excluded patients with abnormal renal function based on the serum creatinine values. Eleven studies met all the criteria. Two Nuclear Medicine physicians independently measured SUV_max_ values in 31 bone locations on the axial and appendicular skeleton, avoiding areas with degenerative changes. The region of interest (ROI) used in this study was 826 mm^3^. The maximum SUV, SUVmax, represents the tracer uptake per voxel. A fixed-size ROI was placed on the selected bone site and SUV_max_ was recorded (Figure 1).

### Technique

60 minutes following the intravenous administration of NaF18, CT transmission images without intravenous contrast was acquired from the vertex to the toes for attenuation correction and anatomic localization. This was followed by a PET emission scan over the same anatomical regions. Imaging was performed in a PET/CT scanner (GE STE 64 slice CT scanner, GE healthcare, Waukesha, WI). A transmission scan (5 mm contiguous axial cuts) was obtained using an integrated multi-slice helical non-enhanced CT. The acquisition was obtained in time of flight mode at 3 minutes per bed position with a one-slice overlap at the borders of the field of view to avoid artifacts, using 120 kV, 40 mAs, and a 512×512 matrix size, acquiring a field of view (FOV) of 50 mm for CT and 70 mm for attenuation correction in 500 ms. Immediately after and without moving the patient, an emission scan was obtained in 3D mode in 11 beds at 3 minutes per bed over the same anatomical regions. The PET emission scan was corrected using segmented attenuation data of the conventional transmission scan. A Gaussian filtering (6.4 mm) was performed for smoothing of images. The PET images were reconstructed with a standard iterative algorithm (OSEM, two iterative steps, 24 subsets) using GE software release 5.0 VUE Point FX intelligent reconstruction. CT data were reduced to an image matrix of 128×128. FDG and CT images were “hardware” co-registered. The voxel size of the final co-registered PET/CT image was 3.75×3.91×4.25 mm. All images were reformatted into axial, coronal, and sagittal views. A rotating 3D MIP, as well as axial, coronal and sagittal PET images with and without attenuation correction was interpreted. Acquired CT and PET/CT images were reviewed alongside the PET images.

### Statistics

We used the SPSS version 20 to report the descriptive statistics and to draw the boxplot.

## Results

**Figure 2:**
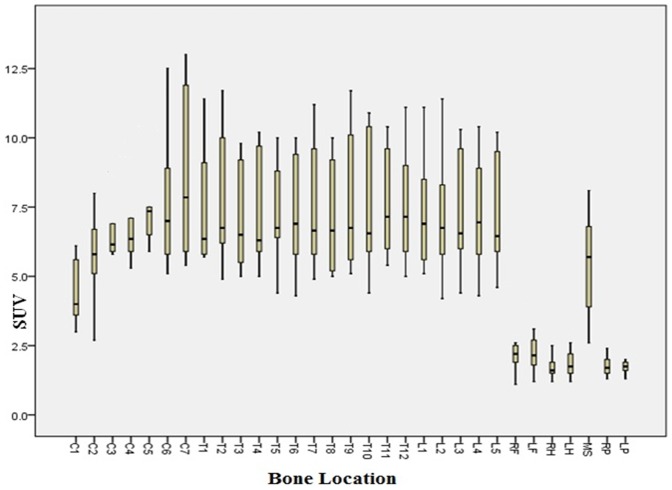
Distribution of SUVmax values of 11 patients and corresponding bone locations (C = cervical, T = thoracic, L = lumbar, RF = right femur, LF = left femur, RH = right humerus, LH = left humerus, MS = mid sternum, RP = right parietal, LP = left parietal). Boxes represent values between 25^th^ and 75^th^ percentiles, horizontal bars inside boxes indicate median, and vertical bars above and below boxes represent 10^th^ and 90^th^ percentiles.

The characteristics of patients are shown in Table 1. The dose of NaF 18 injected range from 9.5–12.6 mCi depending on the weight of the patient. The average normal SUV_max_ values from 11 patients are shown in Table 2. Figure 2 shows the distribution of SUV_max_ values in a boxplot.

**Table 1:** Patient characteristics.

Patient	Age	Reason for NaF18 PET/CT bone scan
1	54	abnormal Right femur, to rule out metastasis
2	50	assess right 7th rib incidental finding
3	66	evaluate for lytic lesions found on CT
4	62	presumed metastasis to bones
5	89	sclerotic lesion on X Ray
6	42	To rule out skeletal coccidioidomycosis
7	63	Status post left total hip arthroplasty with bone pain
8	65	knee joint replacement
9	62	arthropathy of left ankle
10	83	Benign prostatic hyperplasia (BPH)
11	77	elevated PSA

**Table 2:** Mean SUV_max_ of 31 skeletal sites.

Skeletal Site	Mean SUV_max_	Skeletal Site	Mean SUV_max_
**C1**	4.38	**T10**	7.48
**C2**	5.63	**T11**	7.58
**C3**	6.75	**T12**	7.3
**C4**	6.97	**L1**	7.16
**C5**	7.75	**L2**	7.04
**C6**	7.72	**L3**	7.17
**C7**	8.64	**L4**	7.72
**T1**	7.55	**L5**	7.26
**T2**	7.65	**RF**	2.16
**T3**	7.12	**LF**	2.28
**T4**	7.24	**RH**	1.71
**T5**	7.26	**LH**	1.92
**T6**	7.28	**MS**	5.51
**T7**	7.36	**RP**	1.75
**T8**	6.99	**LP**	1.68
**T9**	7.52		

Note: C = cervical, T = thoracic, L = lumbar, RF = right femur, LF = left femur, RH = right humerus, LH = left humerus, MS = mid sternum, RP = right parietal, LP = left parietal.

## Discussion

SUV can be used to quantify NaF-18 PET/CT studies. This is the first study utilizing NaF18-PET/CT bone scan on patients free of cancer and metabolic bone disease. According to our study, various skeletal sites have different normal SUV values. For example, the mean SUVs of the spine are higher than the mean SUVs of the femoral bones. This finding is supported by Puri et al who wrote that there was lower bone blood flow at the proximal femur compared to the spine [19]. The spine is the best site for quantitative assessment of bone metabolism because bone turnover is greater than that observed at other skeletal sites [20]. Vertebral bodies exhibited the highest SUV values in our study. The difference in SUV values of bone segments can also be explained by the bone composition. The humerus has predominantly cortical bone and has a lower level of bone metabolism compared to lumbar spine which is rich in trabecular bone [21]. Another reason may be that the spine, lumbar vertebra in particular, is a primary weight bearing bone in the body and the spine is subjected to mechanical stress. Mechanical stress enhances interleukin 11 expression and this stimulates osteoblast differentiation [22]. As a result, the spine has increased bone turn over and increased osteoblastic activity leads to high SUV uptake.

18F-fluoride ions tend to have greater deposition in the axial skeleton (e.g., vertebrae and pelvis) than in the appendicular skeleton and greater deposition around joints than in the shafts of long bones [15]. Brenner et al measured SUV values from NaF18-PET/CT studies of 33 patients with bone tumors who received treatment [23]. Their mean SUV values were: thoracic spine = 5.9, femur = 1.8, and humerus = 1. The mean SUV values from our study were: thoracic spine (T1–T12) = 7.36, femur (right and left) = 2.22 and humerus (right and left) = 1.82. This shows that SUV values can be different between the normal bones and bones with tumor. Additionally, it must be noted that SUV values also depend on the instrumentation and reconstruction methods. Li et al reported that the mean SUV values in the humerus, tibia and femur are about 15–25% of the value in the spine [24]. In this study, the mean SUVs of the parietal bone, and humerus fall within 25% of spine (C1-L5) SUV = 7.15, except femur, which was 31% of the spine SUV. This proves that, in general, the normal spine has the highest SUV uptake in the body.

On the other hand, non-weight bearing bones such as the parietal bones have the lowest SUV values in our study (Figure 1). The mean SUV_max_ of L5 from a study of twenty women with osteoporosis was 6.92. The mean SUV_max_ of L5 from our study was 7.26 [25]. This is expected because there is decreased osteoblastic activity in osteoporosis, resulting in lower SUV. The component of bone turnover being measured by 18F imaging is osteoblastic activity [25]. On the other hand, SUV uptake can be higher in multiple myeloma (MM) patients, compared to the normal persons because there can be increased reactive osteoblastosis on the periphery of the MM lesions [11]. This further supports the fact that there is a difference in SUV values between the normal skeleton and the ones with metabolic bone diseases and cancers.

In a study of 47 bone tumors, Shin et al. reported a threshold SUV value of 3.7 [26], while Duarte et al determined the SUVmax threshold for differentiation between malignant and benign bone lesions to be 2.5 [27]. Kurdziel et al reported that normal bone should have SUV of 10 or less on NaF18 PET/CT [5]. Currently, there is no consensus on the SUV cutoff in NaF18 studies. Metastasis is highly suspected in vertebra when the abnormally increased activity involves the vertebral bodies in addition to the contiguous pedicle and posterior element or when the configuration is rounded [14]. Patterns associated with benign lesions include a mild degree of abnormal activity, location along the plane of the disc space or at a facet joint, peripheral location, and linear configuration [14]. In screening for metastases, sensitivity is more valuable than specificity because false-negative results can entail serious consequence for patients.

In NaF18-PET/CT exams, precise anatomical location of lesions is possible due to CT correlation, which also increases the specificity of the studies. It is crucial to know the SUV values of different bone sites, to follow the treatment response. Blake et al found that the skull, femur and lumbar spine all have different responses to treatment in osteoporosis [28]. Therapeutic interventions targeting metabolic (e.g., osteoporosis, osteomalacia, primary and secondary hyperparathyroidism, Paget’s disease, renal osteodystrophy), degenerative (e.g., osteoarthritis, degenerative joint disease), traumatic (e.g., osteonecrosis), and neoplastic bone diseases can be assessed with NaF18-PET/CT [29]. Kobayashi et al found that SUV_max_ was associated with osteoarthritis stage [22]. Messa et al evaluated response to therapy in patients with renal osteodystrophy, using NaF18-PET [30]. NaF18-PET/CT is very versatile and its use has been increasing. It has the advantage of being able to image the whole body in one noninvasive exam. SUV measurements to quantify NaF18-PET/CT studies in many medical conditions have been very promising.

## Conclusion

According to our study, various skeletal sites have different normal SUV values. Vertebral bodies tend to show the highest SUV values. SUV values can be different between the normal bones and bones with tumor or metabolic bone disease. SUV can be used to quantify NaF-18 PET/CT studies. If the SUV values of the normal skeleton are known, they can be used in the characterization of bone lesions and in the assessment of treatment response to cancer, osteomyelitis, trauma and metabolic bone diseases.
